# FLLL32 Triggers Caspase-Mediated Apoptotic Cell Death in Human Oral Cancer Cells by Regulating the p38 Pathway

**DOI:** 10.3390/ijms222111860

**Published:** 2021-11-01

**Authors:** Chun-Wen Su, Chun-Yi Chuang, Yi-Tzu Chen, Wei-En Yang, Yi-Ping Pan, Chiao-Wen Lin, Shun-Fa Yang

**Affiliations:** 1 Institute of Medicine, Chung Shan Medical University, Taichung 402, Taiwan; jeff11041986@gmail.com (C.-W.S.); weienyang@gmail.com (W.-E.Y.); ichi110385@gmail.com (Y.-P.P.); 2 Department of Medical Research, Chung Shan Medical University Hospital, Taichung 402, Taiwan; 3 School of Medicine, Chung Shan Medical University, Taichung 402, Taiwan; cyi4602@gmail.com; 4 Department of Otolaryngology, Chung Shan Medical University Hospital, Taichung 402, Taiwan; 5 School of Dentistry, Chung Shan Medical University, Taichung 402, Taiwan; chenyitzu0831@gmail.com; 6 Institute of Oral Sciences, Chung Shan Medical University, Taichung 402, Taiwan; 7 Department of Dentistry, Chung Shan Medical University Hospital, Taichung 402, Taiwan

**Keywords:** oral cancer, FLLL32, apoptosis, HO-1, p38

## Abstract

Oral cancer is the most common oral malignant tumor in Taiwan. Although there exist several methods for treatment, oral cancer still has a poor prognosis and high recurrence. FLLL32, a synthetic analog of curcumin with antitumor activity, is currently known to induce melanoma apoptosis and inhibit tumor growth in various cancers. However, few studies have examined the mechanisms of FLLL32 in oral cancer. In this study, we explore whether FLLL32 induces apoptosis in oral cancer. We determined that FLLL32 can inhibit the cell viability of oral cancer. Next, we analyzed the effect of FLLL32 on the cell cycle of oral cancer cells and observed that the proportion of cells in the G2/M phase was increased. Additionally, annexin-V/PI double staining revealed that FLLL32 induced apoptosis in oral cancer cells. Data from the Human Apoptosis Array revealed that FLLL32 increases the expression of cleaved caspase-3 and heme oxygenase-1 (HO-1). FLLL32 activates proteins such as caspase-8, caspase-9, caspase-3, PARP, and mitogen-activated protein kinases (MAPKs) in apoptosis-related molecular mechanisms. Moreover, by using MAPK inhibitors, we suggest that FLLL32 induces the apoptosis of oral cancer cells through the p38 MAPK signaling pathway. In conclusion, our findings suggest that FLLL32 is a potential therapeutic agent for oral cancer by inducing caspase-dependent apoptosis and HO-1 activation through the p38 pathway. We believe that the activation of HO-1 and the p38 pathway by FLLL32 represent potential targets for further research in oral cancer.

## 1. Introduction

Oral cancer is the most common oral malignant tumor. In 2020, oral cancer ranked sixth among all cancer deaths and fourth among male cancer deaths in Taiwan. About 90% of oral cancers are oral squamous cell carcinoma (OSCC), and the most common sites are the tongue, the upper and lower gums, the floor of the mouth, the upper jaw, and the buccal mucosa [1]. Oral squamous cell carcinoma accounts for about 1–2% of all human malignancies, and it is considered to have a high potential for invasion and metastasis [2]. The most common risk factors for oral cancer include betel nut chewing, smoking, drinking, viruses, radiation, eating habits, and other factors [3,4]. Although there are many treatments for oral cancer, including surgery, radiation therapy, chemotherapy, and targeted therapy, oral cancer still has delayed clinical detection, without specific biomarkers for the disease, along with a poor prognosis and high recurrence [5]. The loss of control of primary tumors and lymph node metastasis are the main causes of death in patients with oral cancer.

Curcumin is a phenolic compound extracted from turmeric reported to have antioxidant, antimicrobial, anticancer, and liver and kidney protection effects [6,7]. Curcumin also has many anticancer properties by regulating cell growth, apoptosis, migration, invasion, and EMT (epithelial-to-mesenchymal transition) in several types of cancer [8,9,10]. However, the application of curcumin is still limited by its low bioavailability and rapid metabolism [11]. FLLL32 is a synthetic analog of curcumin, which replaces the two hydrogens on the middle carbon with spiro-cycloalkyl rings to generate a diketo form. Such a chemical modification can prevent the enolization of FLLL32, thereby making it more stable [12]. In previous studies, FLLL32 showed better cytotoxicity in colorectal cancer, glioblastoma, multiple myeloma, and liver cancer cells compared with curcumin [12]. In addition, FLLL32 effectively inhibited the tumor growth ability of breast cancer and pancreatic cancer cells [13]. In a melanoma study, FLLL32 was found to inhibit the growth of melanoma cells and induce cancer cell apoptosis [13]. Moreover, FLLL32 not only promoted the apoptosis of colon cancer cells but also activated an immune response to immune-tolerant cancer cells [14]. However, few studies have examined the mechanisms of FLLL32 in oral cancer. In this study, we aimed to explore the anticancer effects and molecular mechanisms of FLLL32 in oral cancer.

## 2. Results

### 2.1. FLLL32 Inhibits Cell Viability in Oral Cancer Cells

**Figure 1:**
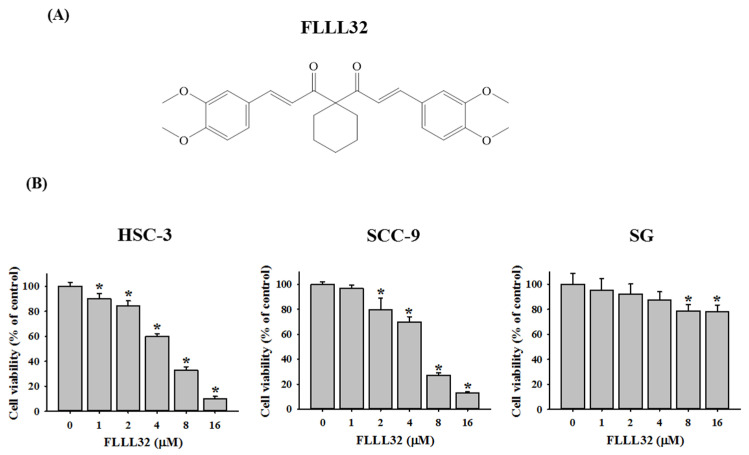
FLLL32 inhibits cell viability in oral cancer cells. (**A**) The chemical structure of FLLL32. (**B**) The cell viabilities of HSC-3, SCC-9, and SG were measured by an MTT assay after treatment with 0, 1, 2, 4, 8, or 16 μM of FLLL32 for 24 h. Data are presented as the mean ± SD, and all experiments were independently repeated at least three times; * *p* < 0.05 compared with the vehicle group.

The structure of FLLL32 is shown in Figure 1A. The effects of FLLL32 on the cell proliferation of HSC-3, SCC-9, and SG were evaluated by an MTT assay with different concentrations of FLLL32 (0, 1, 2, 4, 8, and 16 μM). As shown in Figure 1B, FLLL32 significantly suppressed the cell viabilities of HSC-3 and SCC-9 after FLLL32 treatment for 24 h. Treatment with 16 μM of FLLL32 for 24 h resulted in an approximately 90% reduction in HSC-3 and SCC-9 cell lines. In addition, we tested the effect of FLLL32 on the cell viability of HSC-3 by lengthening the treatment time to 96 h with lower doses. The results showed that the cell viability of FLLL32 at concentrations of 1 μM, 2 μM, and 4 μM was 85.0%, 43.2%, and 2.3%, respectively (Figure S1). However, FLLL32 treatment (16 μM) only reduced human oral gingival SG cells by approximately 20%.

### 2.2. FLLL32 Causes G2/M Arrest and Induces Cell Apoptosis in Oral Cancer Cells

**Figure 2:**
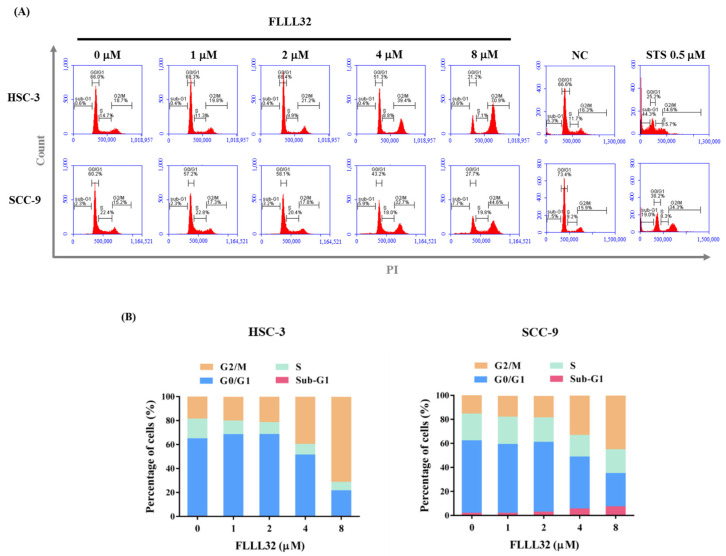
FLLL32 causes G2/M arrest in oral cancer cells. (**A**) HSC-3 and SCC-9 cells were treated with 0, 1, 2, 4, or 8 μM FLLL32 for 24 h and then stained with PI. The distribution of each cell phase was analyzed by flow cytometry. NC: negative control; STS: staurosporine. (**B**) The quantitative cell-cycle phase distribution indicated that G2/M arrest was caused by FLLL32 in HSC-3 and SCC-9 cells in a dose-dependent manner.

**Figure 3:**
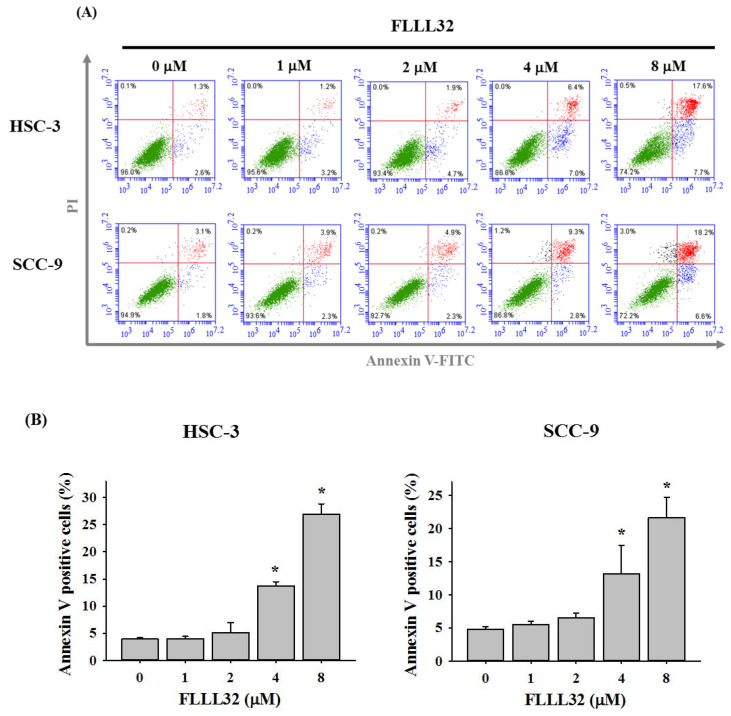
FLLL32 induces cell apoptosis in oral cancer cells. (**A**) HSC-3 and SCC-9 cells were treated with 0, 1, 2, 4, or 8 μM FLLL32 for 24 h and subjected to flow cytometry after annexin V–FITC/PI staining. (**B**) The quantitative percentage of apoptotic cells, including early apoptotic and late apoptotic cells, was increased by FLLL32 in HSC-3 and SCC-9 cells in a dose-dependent manner. Data are presented as the mean ± SD, and all experiments were independently repeated at least three times; * *p* < 0.05 compared with the vehicle group.

Next, to determine the cell growth inhibition effect of FLLL32, HSC-3 and SCC-9 cells were stained with PI and analyzed for DNA content by flow cytometry following FLLL32 treatment (0, 1, 2, 4, and 8 μM). As shown in Figure 2A,B, FLLL32 significantly increased the proportion of G2/M-phase cells in both HSC-3 and SCC-9 cell lines. To verify whether the growth inhibition effect by FLLL32 was caused by cell apoptosis, annexin V–FITC/PI double staining was applied. As shown in Figure 3A,B, FLLL32 significantly increased the proportion of apoptotic cells after 24 h.

### 2.3. The Increase in HO-1 Is Involved in the Apoptosis Regulated by FLLL32

**Figure 4:**
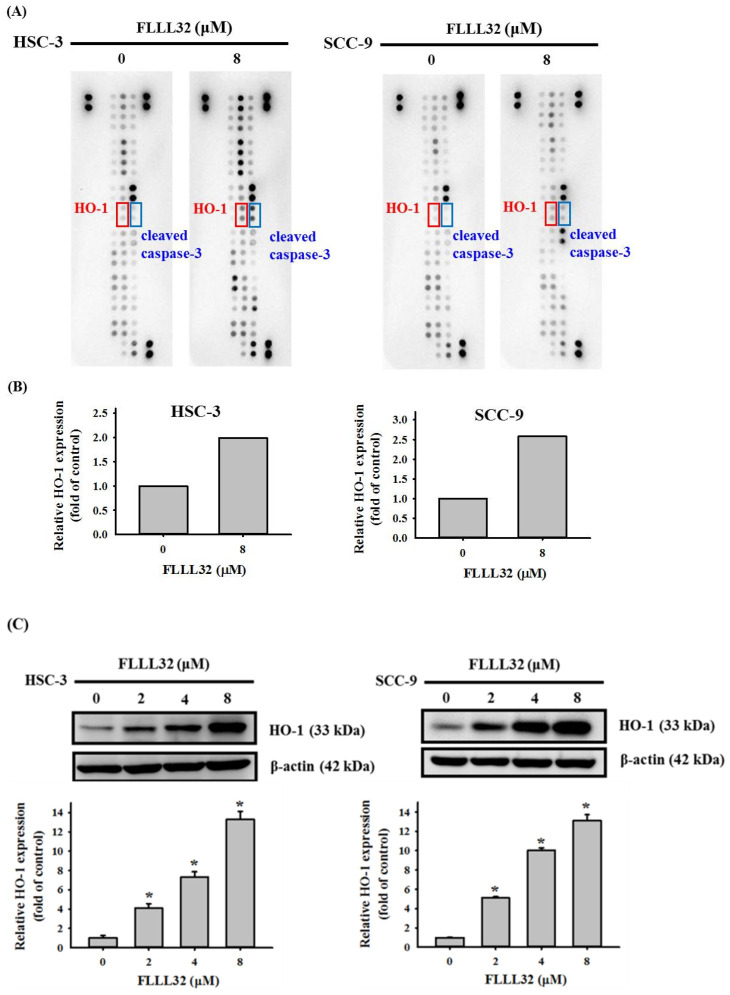
An increase in HO-1 is involved in the apoptosis regulated by FLLL32. (**A**) Representative images of the Human Apoptosis Array (R&D System) including 35 apoptosis-related proteins are shown for HSC-3 and SCC-9 cells after FLLL32 (8 μM) treatment for 24 h. (**B**) The protein expression of HO-1 from the Human Apoptosis Array was quantitated using a densitometer, represented as the fold change compared to the vehicle group. (**C**) The protein expression of HO-1 after FLLL32 treatment (0, 2, 4, and 8 μM) was also confirmed by Western blot and quantified by Image J. β-actin protein levels were used to adjust the quantitative results of protein levels; * *p* < 0.05 compared with the vehicle group.

In order to understand the mechanism of FLLL32 in inducing the apoptosis of oral cancer cells, the Human Apoptosis Array was used to analyze 35 proteins related to the apoptosis pathway. HSC-3 and SCC-9 cells were treated with FLLL32 (8 μM) for 24 h, and the Human Apoptosis Array was performed to observe the expression of apoptosis-related proteins. As shown in Figure 4A,B, the expression of cleaved caspase-3 and HO-1 proteins increased in both oral cancer cell lines after treatment with FLLL32 (8 μM). These results were confirmed by Western blot, which suggested that HO-1 protein levels were significantly increased in both oral cancer cell lines (Figure 4C).

### 2.4. FLLL32-Induced Activation of Caspase-3, -8, -9, and PARP in Oral Cancer Cells

**Figure 5:**
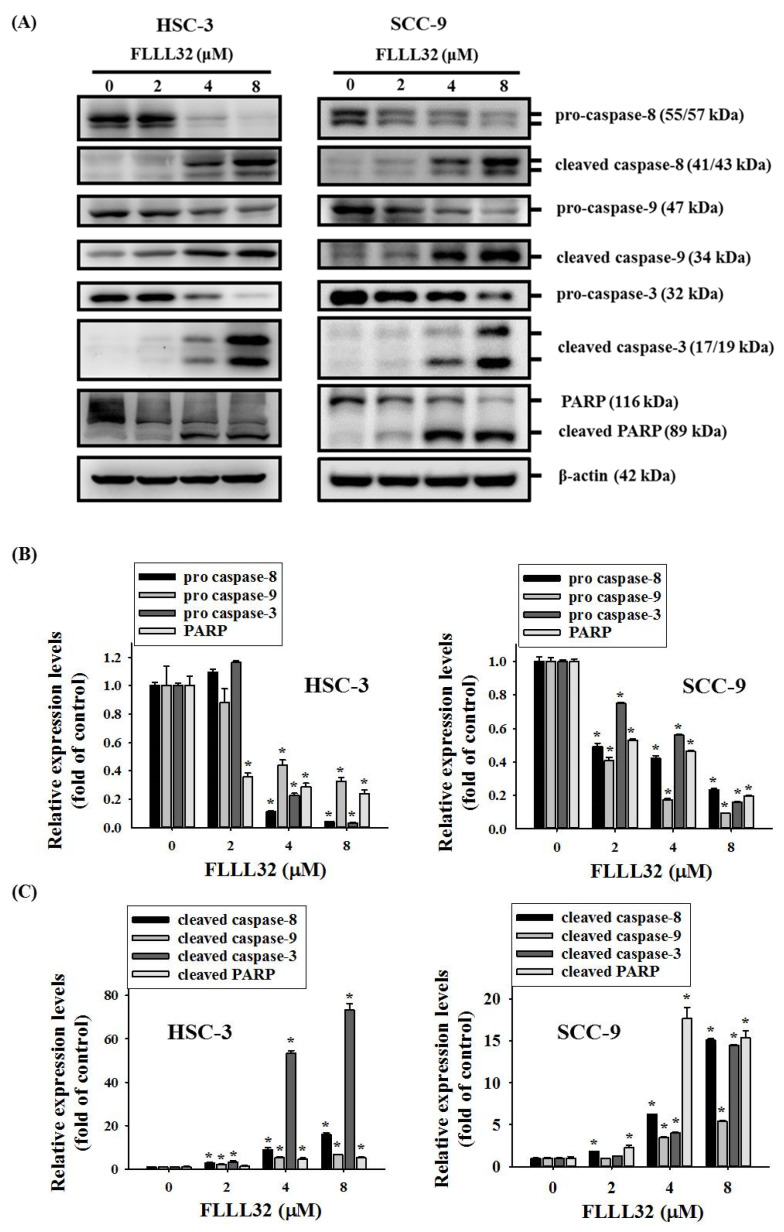
FLLL32 induces the activation of caspase-8, -9, -3, and PARP in oral cancer cells. (**A**) HSC-3 and SCC-9 cells were treated with FLLL32 (0, 2, 4, or 8 μM) for 24 h, and the protein levels of pro- and cleaved caspase-8, -9, -3, and PARP were analyzed by Western blot. The β-actin protein was used as a loading control. (**B**) Quantitative results of pro-caspase-8, -9, -3, and PARP from (**A**). (**C**) Quantitative results of cleaved caspase-8, -9, -3, and PARP from (**A**). β-actin protein levels were used to adjust the quantitative results of protein levels; * *p* < 0.05 compared with the vehicle group.

To further explore the mechanism of FLLL32-induced apoptosis, oral cancer cells (HSC-3 and SCC-9) were treated with different concentrations of FLLL32 (0, 2, 4, and 8 μM) for 24 h, and the changes in apoptosis-related proteins were evaluated by Western blot. As shown in Figure 5A–C, FLLL32 induced the degradation of pro-caspase-8, pro-caspase-9, pro-caspase-3, and PARP in a dose-dependent manner, and it significantly activated the expression of cleaved caspase-8, cleaved caspase-9, cleaved caspase-3, and cleaved PARP. These results confirm that FLLL32 can stimulate the expression of cleaved caspase-8 and cleaved caspase-9 through the extrinsic death receptor pathway and intrinsic mitochondrial pathway, subsequently cleaving and activating the downstream caspase-3 and PARP to induce apoptosis.

### 2.5. Involvement of p38 in FLLL32-Induced Activation of Caspase-3 and HO-1

**Figure 6:**
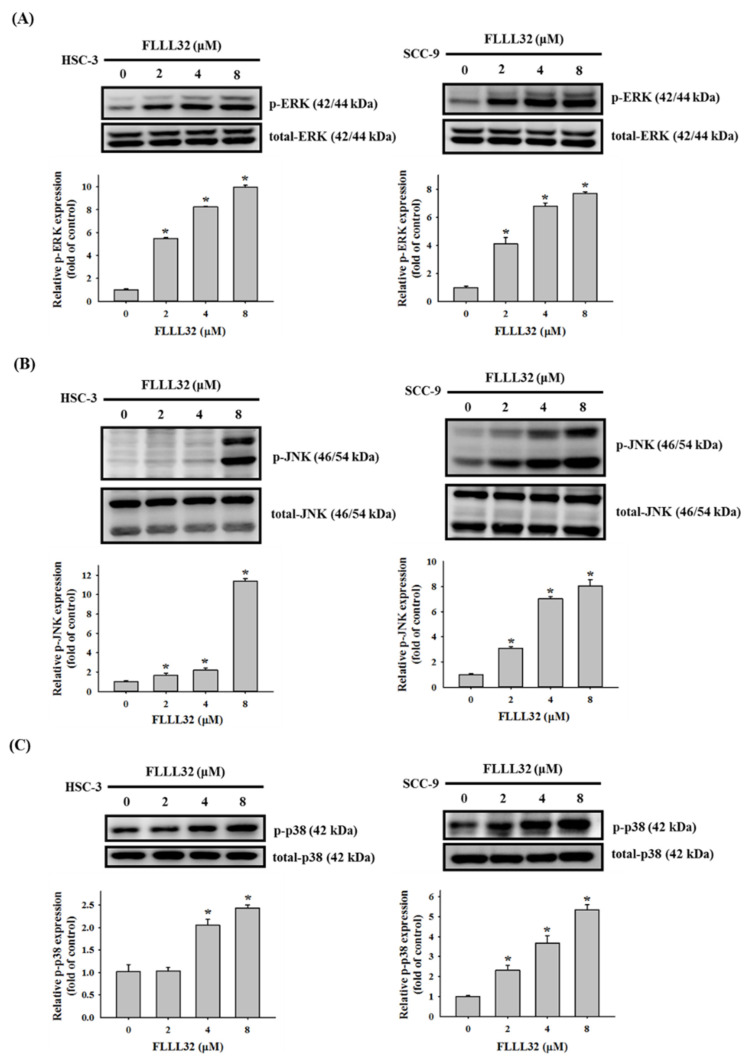
FLLL32 induces the activation of p-ERK, p-JNK, and p-p38 in oral cancer cells. HSC-3 and SCC-9 cells were treated with FLLL32 (0, 2, 4, or 8 μM) for 6 h, and the protein levels were analyzed by Western blot and then quantified by Image J. (**A**) Protein levels of p-ERK and total ERK. (**B**) Protein levels of p-JNK and total JNK. (**C**) Protein levels of p-p38 and total p38. The total protein levels of ERK, JNK, and p38 were used to adjust the quantitative results of protein levels; * *p* < 0.05 compared with the vehicle group.

Previous studies have shown that the MAPK signaling pathway is involved in the apoptosis mechanism induced by curcumin in a variety of cancer models [15]. Therefore, we further explored whether the FLLL32-induced apoptosis of oral cancer cells could be regulated by MAPK proteins. Oral cancer cells (HSC-3 and SCC-9) were treated with different concentrations of FLLL32 (0, 2, 4, and 8 μM) for 6 h, and the changes in MAPK proteins were observed by Western blot. As shown in Figure 6A–C, FLLL32 increased the protein expression of p-ERK, p-JNK, and p-p38 in HSC-3 and SCC-9 cells in a dose-dependent manner. Therefore, we suggest that FLLL32 may regulate cell apoptosis through MAPK proteins, including p-ERK, p-JNK, and p-p38.

**Figure 7:**
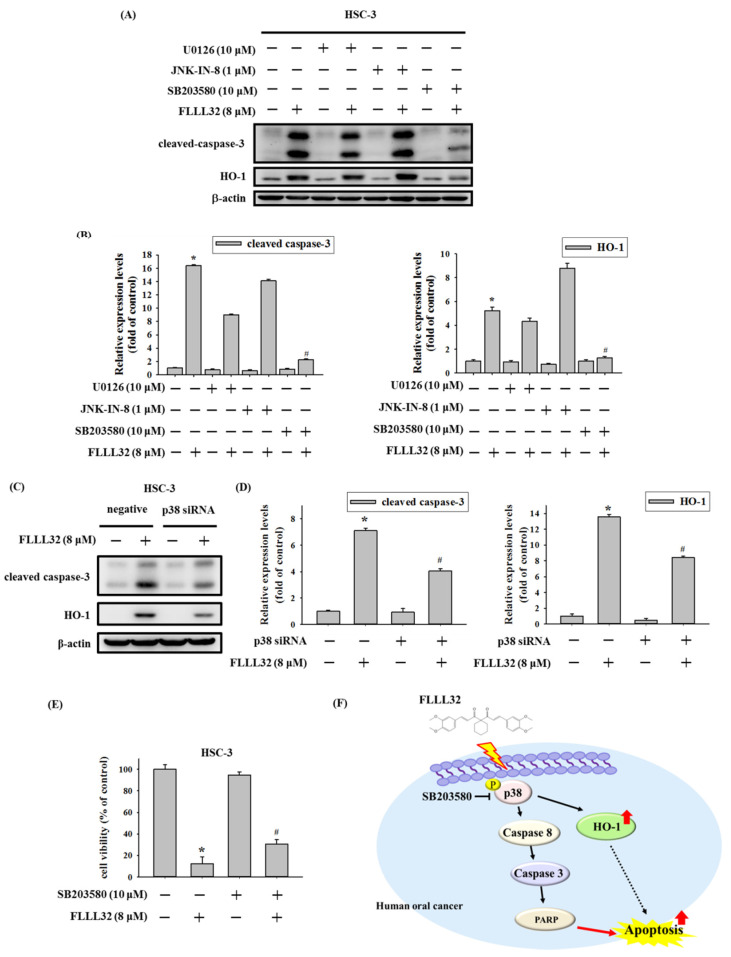
Involvement of p38 in FLLL32-induced activation of caspase-3 and HO-1. (**A**) HSC-3 cells were pretreated with U0126 (10 μM), JNK-IN-8 (1 μM), or SB203580 (10 μM) for 2 h, before treatment with FLLL32 (8 μM) for 24 h. The protein levels of cleaved caspase-3 and HO-1 were analyzed by Western blot. (**B**) Quantitative results of the protein levels of cleaved caspase-3 and HO-1 from (**A**). β-actin protein levels were used to adjust the quantitative results of protein levels. (**C**) The combined effect of p38 siRNA and FLLL32 (8 μM) on cleaved caspase-3 and HO-1 was analyzed by Western blot. (**D**) Quantitative results of the protein levels of cleaved caspase-3 and HO-1 from (**C**). β-actin protein levels were used to adjust the quantitative results of protein levels. (**E**) The combined effect of SB203580 (10 μM) and FLLL32 (8 μM) on cell viability; * *p* < 0.05 compared with the vehicle group; ^#^ *p* < 0.05 compared to the FLLL32-only treated group. (**F**) Proposed model for the anticancer effects of FLLL32 in oral cancer cells. In oral cancer cells, curcumin analog FLLL32 can induce cell apoptosis by activating caspase-3, PARP, and HO-1 through the p38 pathway.

In order to verify whether FLLL32 regulates caspase-induced apoptosis through the MAPK pathway, HSC-3 cells were treated with MAPK pathway inhibitors U0126 (10 μM), JNK-IN-8 (1 μM), and SB203580 (10 μM) for 2 h, followed by treatment with FLLL32 (8 μM) for 24 h; we then observed apoptosis-related proteins and HO-1 by Western blot. As shown in Figure 7A,B, only the p38 inhibitor (SB203580), but not the ERK (U0126) and JNK (JNK-IN-8) inhibitors, could suppress the FLLL32-induced protein expression of cleaved caspase-3 and HO-1. We further used p38 siRNA to confirm our findings with SB203580. As shown in Figure 7C,D, treatment with p38 siRNA also reduced the expression of cleaved caspase-3 and HO-1 activated by FLLL32. In addition, FLLL32 treatment suppressed the protein level of c-Jun, a downstream regulator of the p38 pathway (Figure S2). Next, we tested the effect of the p38 pathway on FLLL32 in inhibiting cell viability. The results revealed that the inhibition of the p38 pathway by SB203580 could reverse the cell viability reduction induced by FLLL32 (Figure 7E). Moreover, data from immunocytochemistry suggested that FLLL32 treatment promoted the nuclear translocation of p38 (Figure S3), which may have been due to DNA damage, as reported in previous studies [16]. On the basis of the above results, we suggest that FLLL32 regulates caspase-mediated apoptosis and HO-1 activation through the p38 signaling pathway (Figure 7F).

## 3. Discussion

Previous studies have pointed out that curcumin has many anticancer properties, e.g., inhibiting tumor differentiation, growth, and metastasis by regulating various transcription factors, growth factors, cytokines, and protein kinases [17]. Although curcumin has such varied anticancer effects, its use is still limited due to its poor water solubility, bioavailability, and chemical stability [18]. According to a study, the bioavailability of oral curcumin is only 1–2% [19]. Therefore, many curcumin analogs have been developed for use in anticancer research. For example, diphenyl difluoroketone (EF-24), with higher oral bioavailability (60%) than curcumin, was reported to trigger cell apoptosis in human acute myeloid leukemia cells [20,21]. In addition, L48H37, with a more stable structure than curcumin, can inhibit the migration and invasion ability of human osteosarcoma [22]. FLLL32 has better cytotoxicity than curcumin in colorectal cancer, glioblastoma, multiple myeloma, and liver cancer [12]. In addition, FLLL32 inhibited cell viability and tumorsphere formation capacity in stem cell-like human colon cancer cells [23]. A combination of FLLL32 and cisplatin activated the sensitivity of head and neck cancer cells to cisplatin [24]. In this study, results from the MTT assay showed that FLLL32 significantly suppressed cell viability via inducing apoptosis in HSC-3 and SCC-9 cells after treatment with different concentrations of FLLL32 (0, 1, 2, 4, 8, and 16 μM). Similar results can also be seen in different types of cancer, whereby FLLL32 treatment induced apoptosis in renal cell carcinoma and melanoma [25].

Cell-cycle checkpoints exist at three stages: the G1/S checkpoint, the G2/M checkpoint, and the spindle assembly checkpoint [26]. These checkpoints can prevent DNA damage and ensure complete DNA replication for mitosis. DNA damage can also lead to cell-cycle arrest, allowing the DNA to undergo DNA repair before replication or mitosis. If the damage cannot be repaired, the cell undergoes apoptosis [27]. Each anticancer drug has a different effect, and the cell arrest caused by anticancer drugs also varies from cancer to cancer. Previous studies have suggested that curcumin caused G1/S and G2/M phase arrest, followed by apoptosis, in human osteosarcoma cells [28]. In head and neck squamous cell carcinoma, WZ37, a curcumin analog, induced G2/M arrest and apoptosis via the oxidant-sensitive Akt/mTOR pathway [29]. A recent study indicated that the curcumin analog DMC caused cell-cycle arrest in the G2/M phase and induced apoptosis in oral squamous cell carcinoma [30]. In order to explore the effects of FLLL32 on the cell cycle and growth of oral cancer cells, HSC-3 and SCC-9 cells were treated with FLLL32, before using PI staining to observe their DNA content. In the present study, FLLL32 caused G2/M phase arrest and inhibited cell growth in both oral cancer cell lines (HSC-3 and SCC-9).

Type 1 heme oxygenase (heme oxygenase-1, HO-1), also known as heat-shock protein 32 (Hsp32), is produced when cells are exposed to hydrogen peroxide, ultraviolet radiation, cytokines, or endotoxins [31]. The role of HO-1 varies in different cancers. In non-small-cell lung cancer, the overexpression of HO-1 promotes tumor invasion and increases the risk of adverse clinical outcomes [32]. In rhabdomyosarcoma, HO-1 expression was increased in cancer cell lines and clinical primary tumors with an aggressive phenotype [33]. In contrast, the overexpression of HO-1 in breast cancer can inhibit cell proliferation [34]. In oral squamous cell carcinoma tissue, a high level of HO-1 is negatively correlated with lymph node metastasis and positively correlated with well-differentiated cancer [35]. Our previous study also suggested that the curcumin analog DMC increased HO-1 expression and induced the apoptosis of oral squamous cell carcinoma cells [30]. In this study, we observed that FLLL32 induced the expression of the HO-1 protein in oral cancer cells (HSC-3 and SCC-9), according to the Human Apoptosis Array and Western blot. On the basis of the above results, HO-1 plays an important role in the regulation of cell apoptosis in oral cancer, but the relevant mechanism still needs to be further clarified.

The MAPK pathway is an important signal pathway that regulates message transmission from outside the cell membrane to the nucleus, and it is closely related to cell proliferation, survival, differentiation, apoptosis, and other physiological processes [36]. Previous studies have pointed out that curcumin and its analogs promoted the apoptosis of cancer cells through the MAPK pathway and downstream caspase activation in several types of cancer [22,37,38]. In oral cancer, tetrahydrocurcumin (THC), a metabolite of curcumin, promoted G2/M arrest and apoptosis via the activation of the p38 pathway [39], and the curcumin analog DMC could also induce G2/M arrest and apoptosis through the p38 pathway. In this study, data from Western blot analysis showed that FLLL32 activated the expression of p-ERK, p-JNK, and p-p38. Further using MAPK inhibitors, we demonstrated that FLLL32 regulated caspase-dependent apoptosis and HO-1 activation through the p38 pathway [30]. Activation of the ERK pathway has been reported to be associated with the progression of cancer, including the promotion of migration, invasion, and EMT [40,41]. However, the activation of ERK can also induce cell apoptosis by regulating mitochondrial cytochrome c release and caspase-8 activation [42]. In our previous studies, many anticancer compounds were found to activate p-ERK and induce apoptosis in different types of cancer [38,43]. The activation of ERK can also reportedly induce cell-cycle arrest, which may cause apoptosis [44]. In another study, treatment with FLLL32 did not affect the expression of p-ERK in osteosarcoma [45] but could promote the activation of p-ERK in melanoma [46]. Interestingly, FLLL32 is also used as a STAT3 inhibitor, whereby it decreases STAT3 DNA-binding activity by interacting with its SH2 domain [13]. In osteosarcoma, FLLL32 treatment decreased p-STAT3 and total STAT3 expression, as well as promoted caspase-3-dependent apoptosis [45]. Inhibition of the STAT3 pathway also reportedly induced cell-cycle arrest and apoptosis in colorectal cancer cells [47]. However, in this study, we did not explore the role of the STAT3 pathway in FLLL32-mediated apoptosis. Therefore, the relationship between the STAT3 pathway and oral cancer cell apoptosis remains to be further confirmed. A limitation of this study is that we did not use animal models to test the effect of FLLL32 on tumor growth. Therefore, the ability of FLLL32 to inhibit tumor growth in vivo is worthy of further research.

## 4. Materials and Methods

### 4.1. Cell and Cell Culture

Human oral squamous cell carcinoma cell line HSC-3 was cultured in MEM medium supplemented with 10% fetal bovine serum (FBS) and 1% penicillin/streptomycin. Human oral squamous cell carcinoma cell line SCC-9 was cultured in DMEM/F12 medium supplemented with 10% FBS, 1% penicillin/streptomycin, 400 ng/mL of hydrocortisone, and 0.1 mM of nonessential amino acids (NEAAs; Life Technologies, Carlsbad, CA, USA). All cell cultures were maintained at 37 °C in a humidified atmosphere of 5% CO_2_.

### 4.2. Microculture Tetrazolium Colorimetric (MTT) Assay

The human oral squamous cell carcinoma cell lines HSC-3 (7 × 10^4^ cells) and SCC-9 (8 × 10^4^ cells) were seeded in 24-well plates and placed in a 37 °C 5% CO_2_ cell incubator for about 16 h. The next day, after removing the old culture medium and washing the cells with 1× PBS, a fresh medium containing FLLL32 was added to the cells for 24 h. After 24 h, the medium was removed, and the MTT reagent was added to the cells for 4 h. Finally, the blue formazan crystals were dissolved in isopropanol, and absorbance was measured at 563 nm using a microplate reader (MQX200; Bio-Tek Instruments, Winooski, VT, USA).

### 4.3. Cell-Cycle Distribution Assay

After FLLL32 treatment for 24 h, the human oral squamous cell carcinoma HSC-3 and SCC-9 cells were fixed in 70% ethanol overnight at −20 °C. The next day, 2 × 10^5^ cells were stained with 0.5 mL of PI/RNase staining buffer for 15 min at room temperature in the dark. The cell-cycle distribution was analyzed using an Accuri C6 Plus flow cytometer (BD Biosciences, San Diego, CA, USA). Cells treated with 0.5 µM staurosporine for 24 h were used as a positive control, whereas cells cultured in complete medium for 24 h were used as a negative control.

### 4.4. Annexin V–FITC Apoptosis Staining Assay

After FLLL32 treatment for 24 h, the human oral squamous cell carcinoma HSC-3 and SCC-9 cells were double-stained with FITC-conjugated annexin V and PI for 15 min in the dark. Three groups (unstained group, PI single-stained group, and annexin V–FITC single-stained group) served as the control groups. The early and late stages of apoptotic cells were analyzed using an Accuri C6 Plus flow cytometer (BD Biosciences, San Diego, CA, USA).

### 4.5. Human Apoptosis Proteome Profiler Array

To identify the apoptosis-related proteins involved in FLLL32-induced apoptosis, the Proteome Profiler Human Array (R&D Systems, Minneapolis, MN, USA) was used for analysis. This kit can simultaneously detect 35 human apoptosis-related proteins. The Image-Pro Plus software was used to quantify the pixel density of the spots. The point density was normalized to the respective reference array points and then normalized to the control.

### 4.6. Protein Extraction and Western Blot Analysis

The human oral squamous cell carcinoma cells were lysed in PRO-PREP protein extraction solution (iNtRON) with protease inhibitor. The total protein (15 μg) was separated by 10–12% SDS-PAGE and then transferred to a polyvinylidene fluoride (PVDF) membrane (Millipore, Belford, MA, USA) for 2 h. The membranes were blocked by 5% BSA for 1 h and then incubated with primary antibodies overnight. The next day, the membranes were incubated with a peroxidase-conjugated secondary antibody for 1 h. Finally, the chemiluminescent signals of the membranes were detected using Luminescent Image Analyzer (LAS 4000 mini, GE Healthcare Bio-Sciences, Pittsburgh, PA, USA). The protein expression was quantified by quantitative software.

### 4.7. Statistical Analysis

All experiments were repeated three times, and the results are presented as the mean ± standard deviation (SD). The Student’s *t*-test was applied to analyze the relationship between two groups using quantitative software (Sigma Plot 14.0). A difference was considered statistically significant at *p* < 0.05.

## 5. Conclusions

In summary, we indicated that the curcumin analog FLLL32 can suppress oral cancer cell viability—as well as induce G2/M cell-cycle arrest and caspase-dependent apoptosis—by activating the p38–HO-1 axis. We suggest that FLLL32 can be used to inhibit tumor growth in combination with clinical treatment to reduce the frequency of radiotherapy and chemotherapy. Moreover, the activation of HO-1 and the p38 pathway by FLLL32 is worthy of further research, as they may represent potential targets for the treatment of oral cancer in the future.

## Data Availability

The data presented in this study are available on request from the corresponding author.

## Supplementary Materials

The following are available online at www.mdpi.com/article/10.3390/ijms222111860/s1.
