# Supplementary material for: FLLL32 Triggers Caspase-Mediated Apoptotic Cell Death in Human Oral Cancer Cells by Regulating the p38 Pathway
Source: Int J Mol Sci. 2021 Nov 1;22(21):11860. doi: 10.3390/ijms222111860 (PMC8584525; doi:10.3390/ijms222111860)
Supplement: Supplementary file 1 [file ijms-22-11860-s001.zip › ijms-1400629-supplementary.pdf]

Figure S1

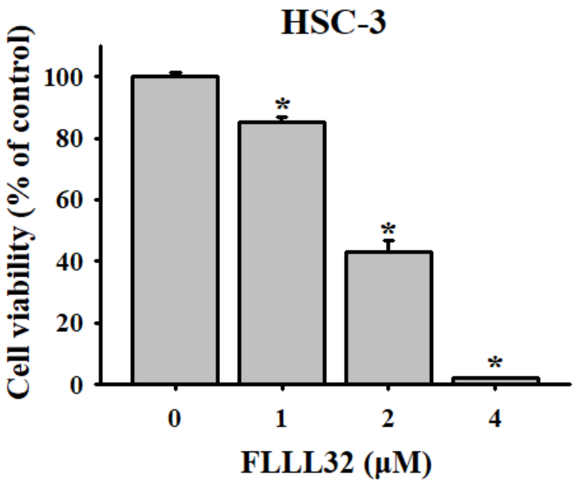

Figure S1. Effect of FLLL32 on cell viability of HSC-3 by lengthening it to 96 hours with lower doses. The results showed that the cell viability of FLLL32 at concentrations of 1 μM, 2 μM, and 4 μM was 85.0%, 43.2%, and 2.3%, respectively. $p<0.05$ compared with vehicle group.

Figure S2

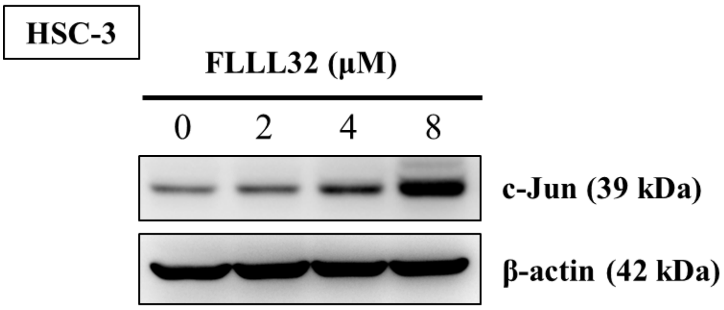

Figure S2. Effect of FLLL32 on p38 pathway downstream regulators c-Jun. The HSC-3 cells were treated with FLLL32 (0, 2, 4, and 8 μM) for 24 h, and the protein levels were analyzed by Western blot. Results suggested that FLLL32 treatment increased expression of c-Jun in a dose-dependent manner.

Figure S3

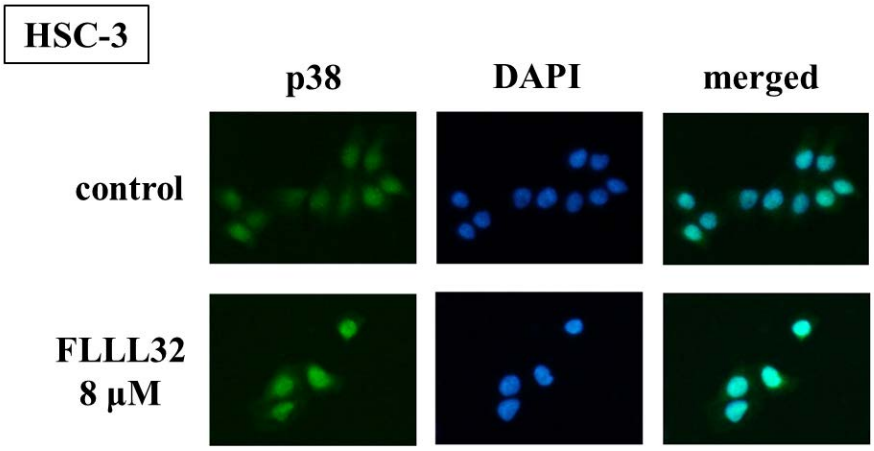

Figure S3. The localization of p38 upon treatment with FLLL32. For immunofluorescence, HSC3 cells were treated with FLLL32 (8 μM) for 6 h**.** The IF staining of p38 (green color) and DAPI staining of nucleus (blue color) were used in HSC-3 cells.
